# Dexmedetomidine versus ketamine for preemptive caudal analgesia in single-level lumbar laminectomy: A randomized controlled trial

**DOI:** 10.1097/MD.0000000000045740

**Published:** 2025-12-12

**Authors:** Mostafa Mohamed Elsayed, Hend Mostafa Ahmed Abosaifa, Gihan Eissa Zahran, Nashwa Mohammed Ibrahiem, Nahla Mohammed Eldeeb, Hend Abdelmonem Elsakhawy, Wafaa Ali Abd Elhadi, Khadiga A. Ismail, Osama Allam Mandour, Mahmoud Mostafa Elshamy, Mohammed Gaber Saad, Ahmed Mosaad Ahmed El Naggar, Ahmed Mohammed Ragab, Mohamed Youssef Hassan, Mohammed Ali A. Majrashi, Samy Mohy Eldin, Tarek Adly Aboubakr, Amira Sobhy Khalil Eldeeb

**Affiliations:** a Department of Anesthesiology, Intensive Care and Pain Management, Faculty of Medicine, Al-Azhar University, Cairo, Egypt; b Department of Anesthesiology, Intensive Care and Pain Management, Faculty of Medicine for Girls, Al-Azhar University, Cairo, Egypt; c Department of Clinical Laboratory Sciences, College of Applied Medical Sciences, Taif University, Taif, Saudi Arabia; d Neurosurgery Department, Faculty of Medicine, Zagazig University, Zagazig, Egypt; e Neurosurgery Department, Faculty of Medicine, Al-Azhar University, Damietta, Egypt; f Division of Pharmacology, Department of Basic Medical Sciences, College of Medicine, University of Jeddah, Jeddah, Saudi Arabia; g Intensive Care Department, Prince Mishari bin Saud Hospital in Baljurashi, Al-Baha Health Cluster, Ministry of Health, Al-Baha, Saudi Arabia; h Intensive Care Department, Fayoum General Hospital, Fayoum, Egypt.

**Keywords:** analgesia, dexmedetomidine, ketamine, lumbar laminectomy, lumbosacral spine

## Abstract

**Background:**

Postoperative pain following spine surgeries remains a persistent challenge for patients, surgeons, and healthcare facilities. A wide range of medications, including ketamine and dexmedetomidine, are used for analgesia in lumbosacral spine surgeries. This study compares the efficacy, hemodynamic effects, and side effects of adding dexmedetomidine versus ketamine as adjuvants to 0.25% bupivacaine for postoperative analgesia following lumbar laminectomy.

**Methods:**

This prospective, randomized, double-blinded study was conducted on 60 adult patients scheduled for elective discectomy/laminectomy of the lumbosacral spine. Patients were categorized into 2 groups: the dexmedetomidine group (1 µg/kg with 20 mL of 0.25% bupivacaine) and the ketamine group (0.5 mg/kg with 20 mL of 0.25% bupivacaine). The primary outcome was postoperative analgesia assessment using the visual analog scale. Secondary outcomes included intraoperative analgesia assessment by monitoring end-tidal sevoflurane levels, hemodynamic parameters, and fentanyl requirements. Postoperative hemodynamic parameters and time to rescue analgesia were also evaluated.

**Results:**

The study demonstrated that dexmedetomidine provided more prolonged analgesia and better immediate postoperative pain control compared to ketamine. This was evidenced by a significantly longer time to rescue analgesia and lower visual analog scale scores in the dexmedetomidine group. Hemodynamic stability was better in the dexmedetomidine group, as evidenced by significantly lower and more stable intraoperative and postoperative heart rate and blood pressure values.

**Conclusion:**

Dexmedetomidine provides superior postoperative analgesia and pain control in the immediate postoperative period compared to ketamine. However, both drugs showed comparable efficacy at later postoperative times, indicating ketamine remains an option for pain management in certain circumstances.

## 1. Introduction

Caudal analgesia, in addition to general anesthesia, is a widely used regional technique for prolonged postoperative analgesia in various surgical procedures. Postoperative pain following lumbosacral spine surgery originates from the manipulation of mechanoreceptors and nociceptors in the vertebrae, nerve roots, dura sleeves, intervertebral discs, facet joint capsules, ligaments, fascia, and muscles. Pain persists beyond the initial surgical injury due to the inflammatory process.^[1]^

The average rate of lumbar spine surgeries among US. Medicare enrollees is 1.7 to 2.2/1000 individuals. Each year, hundreds of thousands of spine surgeries are performed, and it is widely recognized that these patients experience significant postoperative pain.^[2]^ Minimizing postoperative pain in the safest and most effective manner is of utmost importance. Caudal analgesia is a desirable intervention as it prevents central sensitization and provides prolonged analgesia by blocking sensory input from both the primary surgical insult and the secondary inflammatory response when administered preemptively. Additionally, it allows surgeons to assess motor function postoperatively.^[3,4]^

Single-shot CB provides analgesia for 2 to 4 hours, but its duration can be extended with the use of adjuvants such as ketamine, α2-agonists, and opioids.^[5]^ Dexmedetomidine, a selective α2-agonist with a favorable pharmacokinetic profile, is an effective neuraxial adjuvant. Caudal analgesia, in combination with general anesthesia, offers significant pain relief, typically lasting 4 to 6 hours.^[6]^

Dexmedetomidine possesses both analgesic and sedative properties. Although it has minimal respiratory depression – an important advantage – it may cause hypotension and bradycardia, necessitating careful administration. Dexmedetomidine is commonly used for mild to moderate sedation.^[7]^

Ketamine, N-methyl-d-aspartate receptor antagonist, induces dissociative anesthesia and exerts both amnestic and analgesic effects.^[8]^ Studies have shown that the duration of postoperative analgesia is extended when ketamine is added to bupivacaine in CBs. The analgesic effects of caudal epidural ketamine are likely mediated by its interaction with glutamate *N*-methyl-d-aspartate receptors or opioid receptors in the spinal cord.^[9]^

To our knowledge, the comparative analgesic effects of ketamine and dexmedetomidine as preemptive caudal analgesia for lumbosacral spine surgeries have not been well studied. Therefore, this study aimed to evaluate the efficacy of postoperative analgesia in lumbosacral laminectomy by comparing the effects of adding dexmedetomidine (1 µg/kg) versus ketamine (0.5 mg/kg) to bupivacaine 0.25%, with respect to hemodynamic stability and associated adverse effects.

## 2. Materials and methods

This prospective, randomized, double-blinded study was conducted on 60 adult patients undergoing elective single-level lumbar laminectomy at Al-Azhar University Hospitals, Cairo, Egypt. After approval from the ethical committee of Al-Azhar University, Faculty of Medicine in Cairo. Written informed consent was obtained from all patients and the study was conducted over months (May 2024–September 2024).

A pilot study was conducted with 8 patients in each group to determine the sample size. The sample size was calculated to detect a 35% difference in the time to first rescue analgesia. Using Statistical Package for Social Sciences (SPSS Inc., Chicago; version 29.0 for Windows), at power of 0.80, confidence interval of 95 %, significance level of .05 and effect size of 0.35, 30 patients were required in each group.

Patients with age ranging from 18 to 60 years and American Society of Anesthesiologists (ASA) physical status class I or II who were planned for single-level lumbar laminectomy were included in this study. Patients on cardiovascular medications, those with known hypersensitivity to local anesthetics, sacral anomalies, or conditions contraindicating neuraxial blockade, or procedures that require fusion, instrumentation, or multilevel involvement were excluded from the study.

### 2.1. Randomization, blinding, and concealment

Patients were randomly assigned into 2 equal groups using computer-generated numbers and sealed opaque envelopes. Randomization and concealment were performed by a neurosurgery specialist not participating in the study. The dexmedetomidine group received 1 μg/kg of dexmedetomidine combined with 20 mL of 0.25% bupivacaine, while the ketamine group received 0.5 mg/kg of ketamine combined with 20 mL of 0.25% bupivacaine. Both the patients and the attending anesthetists were blinded to group allocation. The sealed envelopes were opened just before the CB by an anesthetist not involved in postoperative assessments. The study drug mixtures were prepared by a separate, independent physician who was also blinded to group allocation and had no further role in patient management or data collection.

### 2.2. Preoperative management

Preoperative data, including age, sex, body mass index, comorbidities such as diabetes and hypertension, anesthesia duration, and surgical time, were recorded. All patients were trained preoperatively to use the visual analog scale (VAS) for pain assessment, which ranges from 0 (no pain) to 10 (worst pain imaginable).

### 2.3. Anaesthetic management

Standard monitoring, including noninvasive blood pressure, pulse oximetry, and electrocardiography, was applied before anesthesia induction. General anesthesia was induced with intravenous fentanyl (2 μg/kg), propofol (2 mg/kg), and rocuronium (0.5 mg/kg) to facilitate endotracheal intubation. Patients were then positioned prone for surgery.

Anesthesia was maintained with sevoflurane (MAC 2%) in a mixture of nitrous oxide and oxygen, along with intermittent boluses of rocuronium (5 mg) administered every 30 to 40 minutes based on neuromuscular monitoring, and fentanyl (25 µg) given every 60 minutes or earlier if heart rate (HR) or mean arterial pressure (MAP) increased by <20% from baseline.

The caudal epidural injection was performed in the prone position under strict aseptic conditions. The loss of resistance technique was used to confirm the epidural space, and correct needle placement was further verified using an image intensifier. The same attending anesthetist performed all CBs, and all surgeries were conducted using the same standardized technique. The total volume of injected medication was identical for all patients.

Intravenous paracetamol (1 g) was administered approximately 30 minutes before the end of surgery and continued every 8 hours for the first 24 hours postoperatively.

The surgical incision was made at least 20 minutes after the CB to ensure adequate drug distribution.

### 2.4. Hemodynamic monitoring and intraoperative management

Hemodynamic parameters, including MAP and HR, were recorded preoperatively and at regular intervals intraoperatively and postoperatively. These baseline values were used for comparison. Hypotension, defined as a 20% decrease in systolic blood pressure from baseline, was treated with ephedrine (6 mg IV). Bradycardia, defined as a 20% decrease in HR from baseline or a HR below 50 beats/minute, was managed with atropine (0.6 mg IV). If MAP or HR increased by 20% from baseline, a fentanyl bolus (0.5 μg/kg IV) was administered as rescue analgesia.

At the end of surgery, neuromuscular blockade was reversed with neostigmine (0.04 mg/kg IV) and atropine (0.01 mg/kg IV). Patients were extubated once they met extubation criteria and were then transferred to the recovery room for further monitoring.

### 2.5. Outcome measurements

The primary outcome was postoperative pain assessment using the VAS at specific time points, including immediately upon full recovery from anesthesia and at 30 minutes, 1, 2, 4, 6, 7, 8, 12, and 24 hours postoperatively by neurosurgery specialists or Anesthesiologist blinded to group allocation. Rescue analgesia with diclofenac (75 mg IV infusion) was administered to patients with a VAS score >4.

The secondary outcomes included intraoperative and postoperative hemodynamic parameters (MAP and HR) recorded at baseline, then at 5, 10, 15, 20, 25, 30, 60, and 90 minutes. End-tidal sevoflurane concentration was also recorded at the same intervals. Total intraoperative fentanyl consumption was documented. The time to first rescue analgesia in minutes was recorded.

### 2.6. Statistical analysis

Statistical analyses were performed using SPSS v29 (IBM, Chicago). Categorical variables were described using their absolute frequencies and compared using chi-square test Continuous numerical data were expressed as mean ± standard deviation. Between-group comparisons for VAS and hemodynamic parameters were conducted using the unpaired Student *t* test and the chi-square test. A *P*-value of <.05 was considered statistically significant. A highly significant difference was present if *P* < .001.

## 3. Results

**Figure 1.**
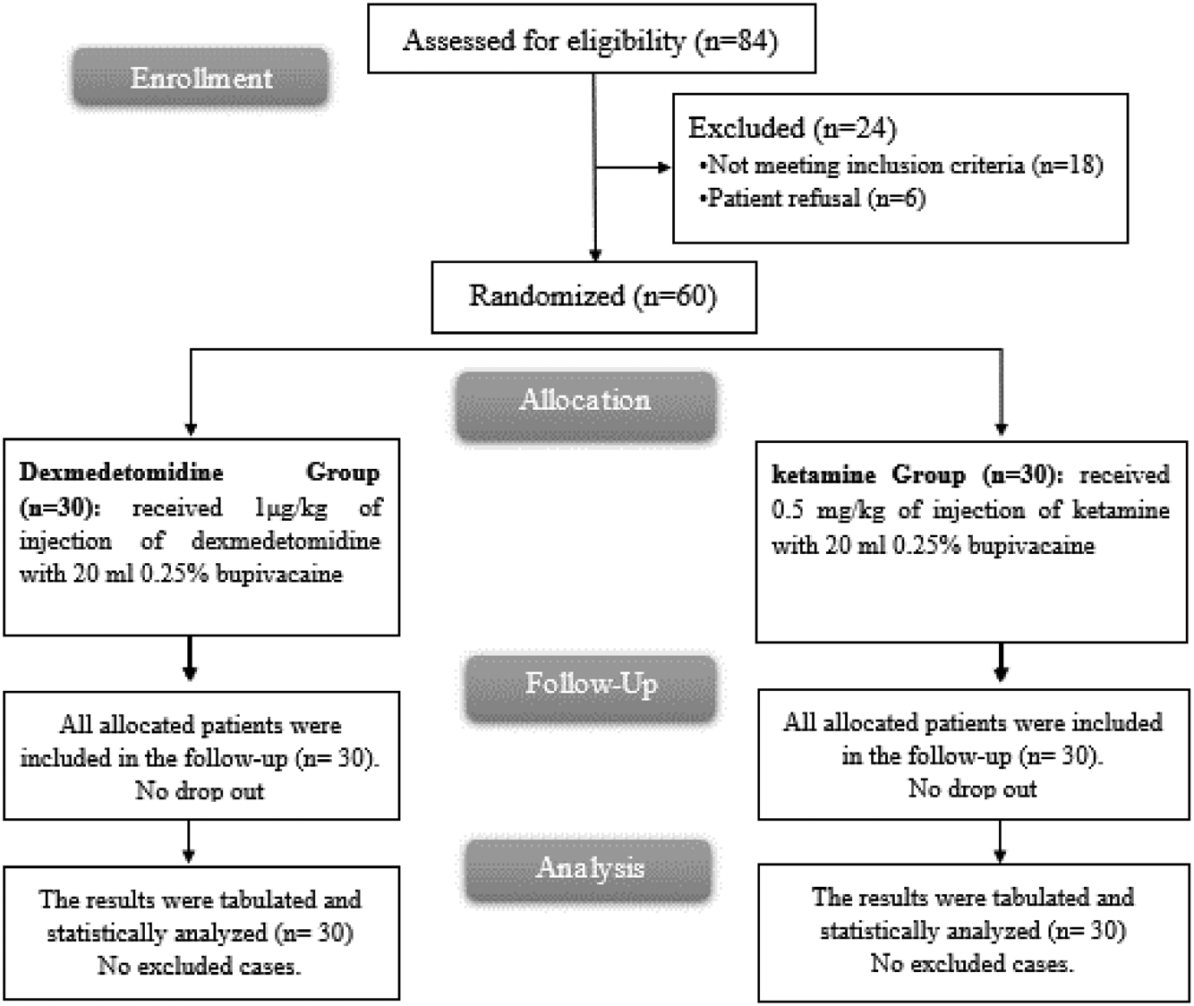
CONSORT flowchart of the enrolled cases.

In this investigation, 84 cases have been evaluated for eligibility, 18 cases did not meet the criteria, and 6 cases refused to participate in the investigation. The remaining cases have been randomly assigned to 2 equal groups, each containing 30 cases. The statistical analysis and follow-up of all allocated cases have been conducted (Fig. 1).

Characteristics of cases, and time of anesthesia were insignificantly dissimilar between the 2 groups (Table 1).

**Table 1:** Cases characteristics, and time of anesthesia of the groups under investigation.

			Dexmedetomidine (n = 30)	Ketamine (n = 30)	*P* value
Age (yr)			43.2 ± 5.76	43.1 ± 6.45	.2415
Sex		Male	12 (40%)	18 (60%)	.657
		Female	18 (60%)	12 (40%)	
BMI (kg/m^2^)			32.2 ± 1.30	35.26 ± 3.31	.53
ASA physical status	I		21 (70%)	14 (46.7%)	.091
	II		9 (30%)	16 (53.3%)	
Duration of anesthesia (min)			158.6 ± 32.52	146.8 ± 25.3219273	.9432

ASA = American Society of Anesthesiologists, BMI = body mass index.

Data are represented as mean ± standard deviation or frequency (%), BMI, ASA.

**Figure 2.**
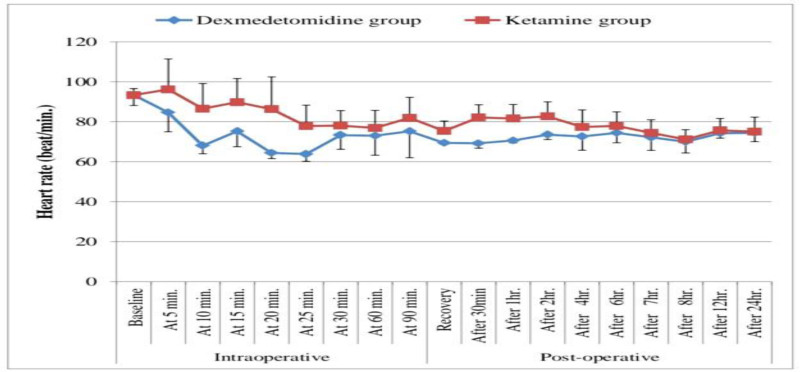
Comparison between dexmedetomidine and ketamine groups according to heart rate.

Intraoperative HR measurements at baseline, after 5 minutes, 10 minutes, 15 minutes, 20 minutes, 25 minutes, 30 minutes, and 1 hour were significantly lower in the dexmedetomidine group than the ketamine group (*P* value < .05). At 90 minutes intraoperatively HR was insignificantly different between dexmedetomidine and ketamine groups (*P* value > .05; Table 2, Fig. 2).

**Table 2:** Intraoperative heart rate of the groups under investigation.

	Heart rate beat/min intraoperative		
	Dexmedetomidine	Ketamine	*P* value
Baseline	93.4 ± 5.29	93.27 ± 3.33	.907
5 min	84.63 ± 9.66	96.13 ± 15.23	<.0001
10 min	68.00 ± 4.07	86.47 ± 12.63	<.0001
15 min	75.20 ± 7.73	89.73 ± 11.84	<.0001
20 min	64.40 ± 2.84	86.27 ± 16.09	<.0001
25 min	63.80 ± 3.66	77.80 ± 10.48	<.001
30 min	73.27 ± 7.07	78.03 ± 7.52	.014
60 min	72.97 ± 9.761	76.83 ± 8.79	.112
90 min	75.17 ± 13.17	81.90 ± 18.27	.107

**Figure 3.**
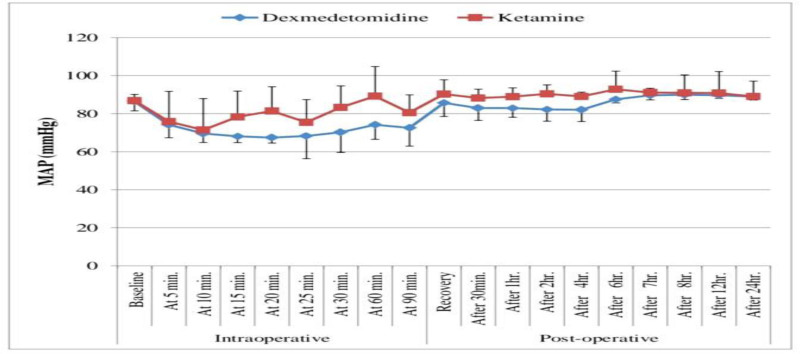
Comparison between dexmedetomidine and ketamine groups according to MAP (mm Hg). MAP = mean arterial pressure.

Intraoperative blood pressure measurements after 15, 20, 25, 30, 60, and 90 minutes were significantly lesser in the dexmedetomidine group compared to the ketamine group (*P* value <.05; Table 3, Fig. 3).

**Table 3:** Intraoperative mean arterial blood pressure of the groups under investigation.

	Mean arterial blood pressure mm Hg intraoperative			
	Dexmedetomidine	Ketamine	*P*-value	
Baseline	86.40 ± 4.88	86.80 ± 3.28		.711
5 min	74.21 ± 6.88	75.80 ± 15.93		.629
10 min	69.53 ± 4.65	71.40 ± 16.52		.554
15 min	68.06 ± 3.26	78.27 ± 13.57		<.0001
20 min	67.40 ± 2.85	81.40 ± 12.75		<.0001
25 min	68.27 ± 11.96	75.43 ± 11.96		.034
30 min	70.20 ± 10.54	83.27 ± 11.29		<.0001
60 min	74.13 ± 7.58	89.17 ± 15.57		<.0001
90 min	72.53 ± 9.56	80.53 ± 9.36		.002

Table 4 showed that a significant reduction has been detected in dexmedetomidine group compared to the ketamine group regarding end-tidal sevoflurane at 10 minutes, 15 minutes, 20 minutes, 25 minutes, 30 minutes, 1 hour, and 90 minutes.

**Table 4:** Intraoperative end-tidal sevoflurane and total fentanyl used intraoperative.

	Dexmedetomidine	Ketamine	*P*-value
End-tidal sevoflurane intraoperative			
Baseline	0	0	**–**
5 min	1.65 ± 0.31	2.27 ± 3.73	.366
10 min	1.00 ± 0	1.4 ± 0.07	<.0001
15 min	0.8 ± 0.08	1.14 ± 0.23	<.0001
20 min	1.00 ± 0.	1.2 ± 0.14	<.0001
25 min	0.8 ± 0	1.04 ± 0.19	<.0001
30 min	0.92 ± 0.	1.19 ± 0.13	<.0001
60min	1.00 ± 0	1.19 ± 0.14	<.0001
90 min	1.00 ± 0	1.18 ± 0.14	<.0001
Total fentanyl	84 ± 2.03	124 ± 30.47	<.0001

A significant reduction has been detected in the total fentanyl consumption in the dexmedetomidine group compared to the ketamine group (*P* < .0001).

HR measurements decreased significantly at immediate postoperative, 30 minutes, 1 hour, 2 hours, 4 hours, 6 hours in dexmedetomidine group compared to the ketamine group (*P* value > .05) with insignificant variance among groups at 7, 8, 12, and 24 hours postoperatively (*P* value > .05). Blood pressure measurements decreased significantly immediate postoperative, 30 minutes, 1 hour, 2 hours, 4 hours, 6 hours, and 7 hours in dexmedetomidine group compared to the ketamine group (*P*-value > .05) with insignificant variance between groups at 8, 12, and 24 hours postoperatively (*P*-value > .05; Table 5, Figs. 2 and 3).

**Table 5:** Heart rate following surgery and blood pressure between both groups.

	Dexmedetomidine	Ketamine	*P*-value
Heart rate beat/min postoperative			
Recovery	69.40 ± 0.81	75.33 ± 5.00	<.0001
30 min	69.2 ± 2.44	82.10 ± 6.37	<.0001
1 h	70.6 ± 1.22	81.6 ± 6.98	<.0001
2 h	73.5 ± 2.41	82.67 ± 7.28	<.0001
4 h	72.6 ± 6.9	77.33 ± 8.58	.022
6 h	74.37 ± 4.94	77.87 ± 7.05	.030
7 h	72.2 ± 6.52	74.377 ± 6.57	.205
8 h	69.93 ± 5.58	71.13 ± 4.86	.378
12 h	74.2 ± 2.44	75.63 ± 6.00	.231
24 h	74.40 ± 4.38	75 ± 7.26	.669
Mean arterial blood pressure mm Hg postoperative			
Recovery	85.66 ± 7.12	90.263 ± 7.51	.018
30 min	82.93 ± 6.5	88.2 ± 4.61	<.001
1 h	82.90 ± 4.77	88.90 ± 4.63	<.001
2 h	82.2 ± 6.18	90.40 ± 4.69	<.001
4 h	82.03 ± 6.18	89.03 ± 2.28	<.001
6 h	87.47 ± 1.85	92.8 ± 9.53	.007
7 h	89.60 ± 2.37	91.07 ± 2.33	.019
8 h	89.93 ± 2.47	90.93 ± 9.47	.448
12 h	89.70 ± 1.72	90.80 ± 11.38	.412
24 h	88.93 ± 1.79	89 ± 8.12	.896

Time to rescue analgesia was significantly extended in the Dexmedetomidine group (476 ± 8 minutes) compared to the ketamine group (342.4 ± 21.2 minutes; Table 6).

**Table 6:** Time to rescue analgesia.

	Dexmedetomidine	Ketamine	*P*-value
Time to rescue analgesia (min)	476 ± 8.14	346.63 ± 33.09	<.001

VAS measurements decreased significantly immediate postoperatively, 30 minutes, 1 hour, 2 hours, and 4 hours, in dexmedetomidine group than the ketamine group (*P*-value < .05) with insignificant variance among groups at 6, 7, 8, 12, and 24 hours postoperatively (*P*-value >.05; Table 7).

**Table 7:** Postoperative VAS.

Time	Dexmedetomidine	Ketamine	*P*-value
Postop	2.67 ± 1.18	1.6 ± 1.45	<.001
30 min	2.50 ± 1.45	2.5 ± 0.97	<.001
1 h	2.23 ± 1.61	2.87 ± 0.86	.012
2 h	2.30 ± 0.91	2.8 ± 0.93	.001
4 h	2.53 ± 0.97	3.23 ± 1.05	<.001
6 h	3.8 ± 0.71	3.8 ± 0.819	.498
7 h	3.23 ± 1.10	3.23 ± 0.94	.102
8 h	3.87 ± 078	3.90 ± 0.80	.956
12 h	3.20 ± 0.66	3.63 ± 0.99	.053
24 h	4.17 ± 0.46	4.27 ± 0.69	.326

VAS = visual analog scale.

## 4. Discussion

Caudal anesthesia is widely used due to its proven efficacy in providing postoperative analgesia; however, it has certain limitations. Patients may experience difficulty walking immediately after surgery due to the bilateral sensory and motor paralysis caused by caudal block (CB), leading to a prolonged recovery period. Another common and potentially severe adverse effect is urinary retention.^[10]^ While adjuvants can enhance intraoperative analgesia and prolong pain relief, they may also contribute to delayed recovery and prolonged sedation.^[11]^

Dexmedetomidine is frequently used for sedation in ICU patients, as well as in those undergoing surgery, cardiac catheterization, or radiographic procedures.^[12]^ In addition to its sedative effects, dexmedetomidine has been shown to reduce anxiety and improve patient comfort during critical care and surgical interventions.^[13,14]^

The major findings of the present study demonstrate that dexmedetomidine significantly prolongs the time to rescue analgesia and reduces fentanyl consumption compared to ketamine. VAS measurements revealed significantly lower pain scores in the dexmedetomidine group immediately postoperatively and at various time points up to 12 hours. Additionally, dexmedetomidine was associated with better hemodynamic stability, characterized by decreased blood pressure, HR, and end-tidal sevoflurane levels compared to ketamine.

The superior postoperative analgesic efficacy of dexmedetomidine over ketamine may be attributed to its mechanism of action. Dexmedetomidine is a highly selective α-2 adrenergic agonist that exerts analgesic effects through central and spinal mechanisms. By activating α-2 adrenergic receptors, dexmedetomidine inhibits norepinephrine release, reducing neuronal excitability and pain transmission. This mechanism likely accounts for the prolonged time to rescue analgesia and the significant reduction in postoperative pain scores observed in the dexmedetomidine group.^[15]^

Conversely, ketamine, provides analgesia by blocking central sensitization and modulating pain pathways. While ketamine is effective for both acute and chronic pain management, its analgesic effects may be more variable than those of dexmedetomidine, particularly in the immediate postoperative period. The shorter time to rescue analgesia and less pronounced pain relief in the ketamine group could be attributed to differences in receptor pharmacology and analgesic mechanisms between the 2 drugs.^[9]^

Our results align with those of Kalappa et al,^[2]^ who concluded that dexmedetomidine is an effective adjuvant to ropivacaine for preemptive caudal epidural analgesia in lumbosacral spine surgeries. Similarly, a recent review of 21 studies involving 1590 pediatric patients found that dexmedetomidine significantly extended the duration of analgesia by 2.5 to 3 times, regardless of the local anesthetic used, particularly at a dose of 1 µg/kg.^[16]^

Ketamine at a dose of 0.5 mg/kg added to 0.15% caudal levobupivacaine has been shown to enhance postoperative analgesia and significantly reduce the need for rescue analgesics in the immediate postoperative period compared to caudal 0.2% levobupivacaine alone.^[17]^ Ram et al^[18]^ demonstrated that the addition of ketamine to 0.25% levobupivacaine resulted in improved analgesia. Previous studies have also reported that caudal ketamine, when combined with bupivacaine or ropivacaine, leads to faster onset and prolonged duration of analgesia compared to local anesthetics alone.^[19]^ Additionally, caudal ketamine at a dose of 0.5 mg/kg provided longer-lasting analgesia than fentanyl at 1 µg/kg when added to bupivacaine (8.23 vs 5.95 hours).^[20]^

Contrary to our findings, Yin et al^[21]^ reported that intraoperative hemodynamics were more stable in the ketamine group than in the dexmedetomidine group. Similarly, El Mourad et al^[22]^ supported the observation that ketamine provides more stable hemodynamic parameters than dexmedetomidine. Additionally, Abbas et al^[23]^ found a significant increase in MAP in the ketamine group compared to dexmedetomidine.

Several limitations must be acknowledged in this study. The study included only ASA I and ASA II patients; therefore, future research should examine high-risk populations to generalize the findings. Additionally, this was a single-center study, limiting its external validity. The study did not assess long-term outcomes, potential adverse effects associated with dexmedetomidine and ketamine, sedation, cognitive function, or patient satisfaction in the immediate postop period. Further investigations using different sedative agents, varying dosages, and additional adjuvants are recommended to expand the understanding of optimal analgesic strategies.

## 5. Conclusion

The study demonstrates that dexmedetomidine provides more prolonged analgesia and better pain control in the immediate duration following surgery compared to ketamine. This was evidenced by a significantly extended time to rescue analgesia and lower pain scores measured by the VAS in the dexmedetomidine group. However, both drugs showed comparable efficacy at certain time points postoperatively, indicating that ketamine remains a viable option for pain management in certain clinical scenarios. Overall, these outcomes highlight the possible benefits of dexmedetomidine in improving postoperative pain management, but further research is warranted to explore its optimal use and possible side impacts in clinical practice.

## Acknowledgments

The authors extend their appreciation to Taif University, Saudi Arabia for supporting this work through project number (TU-DSPP-2024-81).

## Author contributions

**Conceptualization:** Mostafa Mohamed Elsayed, Hend Mostafa Ahmed Abosaifa, Gihan Eissa Zahran, Nashwa Mohammed Ibrahiem, Hend Abdelmonem Elsakhawy, Wafaa Ali Abd Elhadi, Osama Allam Mandour, Mahmoud Mostafa Elshamy, Mohammed Gaber Saad, Ahmed Mosaad Ahmed El Naggar, Tarek Adly Aboubakr, Samy Mohy Eldin, Amira Sobhy Khalil Eldeeb.

**Data collection:** Mostafa Mohamed Elsayed, Hend Mostafa Ahmed Abosaifa, Amira Sobhy Khalil Eldeeb.

**Methodology**: Mostafa Mohamed Elsayed, Hend Mostafa Ahmed Abosaifa, Gihan Eissa Zahran, Nashwa Mohammed Ibrahiem, Hend Abdelmonem Elsakhawy, Mahmoud Mostafa Elshamy, Mohammed Gaber Saad, Ahmed Mosaad Ahmed El Naggar, Khadiga A. Ismail, Ahmed Mohammed Ragab, Mohamed Youssef Hassan, Mohammed Ali A. Majrashi, Amira Sobhy Khalil Eldeeb.

**Resources:** Mostafa Mohamed Elsayed, Wafaa Ali Abd Elhadi, Osama Allam Mandour, Mahmoud Mostafa Elshamy, Mohammed Gaber Saad, Ahmed Mosaad Ahmed El Naggar, Ahmed Mohammed Ragab, Mohamed Youssef Hassan, Mohammed Ali A. Majrashi, Khadiga A. Ismail, Tarek Adly Aboubakr, Samy Mohy Eldin, Amira Sobhy Khalil Eldeeb.

**Statistical analysis**: Mostafa Mohamed Elsayed, Mohammed Gaber Saad.

**Supervision:** Mostafa Mohamed Elsayed, Nashwa Mohammed Ibrahiem, Hend Abdelmonem Elsakhawy, Wafaa Ali Abd Elhadi, Osama Allam Mandour, Mahmoud Mostafa Elshamy, Mohammed Gaber Saad, Ahmed Mosaad Ahmed El Naggar, Ahmed Mohammed Ragab, Mohamed Youssef Hassan, Mohammed Ali A. Majrashi, Amira Sobhy Khalil Eldeeb.

**Writing** – **original draft:** Mostafa Mohamed Elsayed, Hend Mostafa Ahmed Abosaifa, Gihan Eissa Zahran, Mohamed Youssef Hassan, Mohammed Ali A. Majrashi, Amira Sobhy Khalil Eldeeb.

**Writing** – **review & editing:** Mostafa Mohamed Elsayed, Hend Mostafa Ahmed Abosaifa, Gihan Eissa Zahran, Nashwa Mohammed Ibrahiem, Hend Abdelmonem Elsakhawy, Wafaa Ali Abd Elhadi, Osama Allam Mandour, Mahmoud Mostafa Elshamy, Mohammed Gaber Saad, Ahmed Mosaad Ahmed El Naggar, Ahmed Mohammed Ragab, Mohamed Youssef Hassan, Mohammed Ali A. Majrashi, Khadiga A. Ismail, Tarek Adly Aboubakr, Samy Mohy Eldin, Amira Sobhy Khalil Eldeeb.
